# Quantitative assessment of the dispersal of soil‐dwelling oribatid mites via rodents in restored heathlands

**DOI:** 10.1002/ece3.9653

**Published:** 2022-12-27

**Authors:** Andrés A. Salazar‐Fillippo, Bert Teunkens, Herwig Leirs, Jan Frouz, Rudy van Diggelen, Ladislav Miko

**Affiliations:** ^1^ Geobiology Research Group, Department of Biology University of Antwerp Antwerp Belgium; ^2^ Faculty of Science, Institute for Environmental Studies Charles University Prague Czech Republic; ^3^ Evolutionary Ecology Group, Department of Biology University of Antwerp Antwerp Belgium; ^4^ Institute of Soil Biology, Biological Centre Academy of Sciences of the Czech Republic Ceske Budejovice Czech Republic

**Keywords:** heathland succession, oribatid mites, passive dispersal, phoresy, phoretic hosts, rodents, soil mesofauna, topsoil removal

## Abstract

Heathland restoration using topsoil removal requires the re‐colonization of above‐ and belowground communities. Oribatid mites play a key role in the comminution of organic matter and are frequently early colonizers during succession despite their limited mobility. Whereas the assembly of their communities may take decades, passive dispersal likely dominates colonization processes, but especially dispersal via other animals (phoresy) remains poorly studied. Compared to other potential hosts, movement habits and ecology of small rodents may provide dispersal advantages to oribatid communities. We studied dispersal of oribatid mites via small rodents in restored heathland sites of different age. We measured movement patterns of small rodents and extracted mites from their pelts and nests to estimate annual contributions of these rodents to the dispersal of oribatids. We also discussed phoretic estimates reported on other host groups as a reference. Probability estimates of oribatids in pelts and nests showed lower occurrence frequencies compared to other reported phoretic hosts. However, local rodent communities may aid the dispersal of up to 41,000 oribatid mites per year. We highlight the high diversity of oribatid species mounting rodents, unlike strong species‐specific filters reported in other passive pathways. We found that over half (58%) of the oribatid species reproduced asexually and over a third (32%) had a soil‐dwelling lifestyle. We also observed that rodents often travel short distances below 40 m, but occasionally reach distances of up to 100 m, especially in earlier successional stages. *Synthesis and applications*. Our results suggest that rodents may contribute to assembly processes of soil‐dwelling oribatid communities given the slow turnover rate of this group in heathlands. This is accomplished through short‐distance dispersal, and especially in sites at early stages of succession. To our knowledge, we are the first to quantitatively assess the potential dispersal of oribatid mites via rodents.

## Graphical abstract

Heathland restoration using topsoil removal provides an ideal scenario to study colonization processes of soil fauna. We quantitatively assessed passive dispersal of oribatid mites via phoresy using small rodents and compared it to other reported host groups. Our study suggests that small rodents may contribute to the assembly of species‐rich oribatid communities given the slow turnover rate of this group in heathlands.

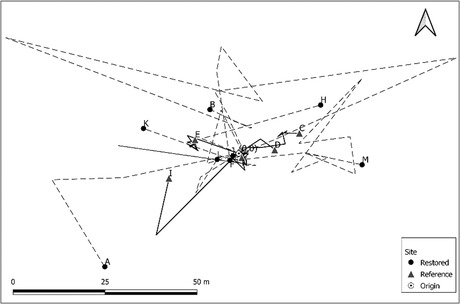

## 1 INTRODUCTION

Restoration of ecosystems has become a crucial challenge to preserve global ecological integrity and human well‐being (Aronson et al., 2020). While this may require only modest interferences in little degraded ecosystems, there are also situations where restoration demands very drastic techniques to reach the target. The conversion of nutrient‐enriched fields into nutrient‐poor ecosystems such as heathlands is such case, and it can be achieved within a reasonable time only with radical approaches such as a complete removal of the fertile top soil (Verhagen et al., 2001). At the same time such topsoil removal has significant trade‐offs, more in particular the requirement that both above‐ and belowground communities must re‐colonize the bare soil to develop into fully functional ecosystems (Hedlund et al., 2003; Korthals et al., 2001).

Vegetation succession is often remarkably fast and shows significant recovery after only a few years (Chytrý et al., 2001; van der Bij et al., 2017), but belowground communities do not automatically follow at the same rate (van der Bij et al., 2018). Such delay is often associated with a slow arrival rate of key terrestrial invertebrates with limited mobility (Kardol et al., 2009; Nielsen et al., 2010; Taylor & Wolters, 2005; Zaitsev et al., 2006). Still, some species of these groups do manage to arrive at early stages of succession, e.g., oribatid mites (Frouz et al., 2001, 2009; Holmstrup et al., 2013; Murvanidze et al., 2008; Wanner & Dunger, 2002). Specimens from this group are widely reported as pioneers in newly created ecosystems (Hågvar et al., 2009; Murvanidze et al., 2013; Skubała & Gulvik, 2005; Wanner & Dunger, 2002), but the assembly of more complete and species‐rich communities may take decades (Salazar‐Fillippo et al., unpublished data; Zaitsev et al., 2006). Given the important role of this group in decomposition, nutrient cycling, and the regulation of microorganisms (Hendrix et al., 1990; Wissuwa et al., 2013), an increased understanding of their hitherto largely unexplored dispersal mechanisms is relevant to gain further insight into soil community functioning with time.

Active dispersal of oribatid mites is generally assumed to be low (Ojala & Huhta, 2001; Skubała & Kafel, 2004). What is clear is that the relative importance of active movement for dispersal is highly species specific and certainly complemented, if not dominated, by passive transport via water, wind, or animals (*phoresy*; Lehmitz et al., 2012). This would explain their early arrival into newly created ecosystems even when potential species pools are in distant locations (Ingimarsdóttir et al., 2012; Lehmitz et al., 2011). Schuppenhauer et al. (2019) reported oribatids floating and surviving submergence, suggesting that aquatic dispersal might also be a feasible pathway in sites surrounded by water bodies. Dispersal via wind is especially efficient for smaller species living in tree habitats (Lehmitz et al., 2011). Moreover, these modes of dispersal both rely strongly on species‐specific resistance to extreme conditions and both result in low colonization success rates (Bailey et al., 2018; Schuppenhauer et al., 2019). This suggests that a development toward species‐rich oribatid communities requires additional dispersal via other pathways. Phoresy is much less studied than dispersal via water and wind and has historically been considered as quantitatively negligible for oribatid dispersal and most likely exclusive to some non‐oribatid mites only (e.g., Prostigmata, Mesostigmata, and Astigmata; Norton, 1980).

More recent studies, however, did find oribatid mites on arthropods (Coulson, 2009; Ermilov & O'connor, 2020; Knee et al., 2013), birds (Krivolutsky & Lebedeva, 2004; Lebedeva, 2007, 2010), amphibians (Beaty et al., 2013; Mendoza‐Roldan et al., 2020), and small mammals (Krawczyk et al., 2015; Miko & Stanko, 1991), suggesting that phoresy could be more relevant for the dispersal of oribatid mites than originally thought. Whether few oribatid species show explicit morphological adaptations for active phoresy (Ermilov & Frolov, 2019b; Norton, 1980), most instances remain uncertain, and “accidental” or “occasional” phoresy might be a more accurate denotation for this type of passive transport. Phoresy via birds is studied most intensively with oribatids reported in up to 66% of the inspected birds, typically ranging from 1 to 2 individuals per host, and in rare exceptions even up to 20 specimens (Krivolutsky & Lebedeva, 2004; Lebedeva, 2007, 2010; Lebedeva & Lebedev, 2008). Reports on dispersal via arthropods, and particularly via passalid beetles (Passalidae), are also fairly common but—with a few exceptions (Knee et al., 2013; Pernek et al., 2008)—lack details on frequencies and abundances, and thus estimations of the real contribution of this group to oribatid dispersal cannot be assessed quantitatively (Ahadiyat & Akrami, 2015; Penttinen et al., 2013; Pernek et al., 2012; Sumangala & Haq, 2001). Herpetofauna remains the least studied with only two atypical reports of anurans with over 80 oribatid mites from the same species (Beaty et al., 2013; Mendoza‐Roldan et al., 2020). Although this clearly suggests deliberate mounting by oribatids, the particular ecology of herpetofauna may have dissuaded researchers from studying the presence of phoretic microarthropods in this group any further in the past (Silva et al., 2020). Small mammals, and specifically rodents, are studied relatively well because they are important dispersers of diseases (Mariën et al., 2018; Olsson et al., 2010) and seeds (Jansen et al., 2012; Wang et al., 2018). The same could be true for oribatids but relatively little is known quantitatively about phoretic interactions between these two groups. Mites have been found both in the pelt and in the nests of small mammals with frequencies of 2%–6% and 44%–70%, respectively (Bukva et al., 1976; Krawczyk et al., 2015; Miko & Stanko, 1991; Vesotskaya & Bulanova‐Zaxvatkina, 1957), but, to our knowledge, quantitative assessments of the dispersal capacity of small mammals for oribatid mites are entirely lacking.

Newly established sites left to spontaneous succession (Prach et al., 2001) provide unique opportunities to study dispersal processes. The present study uses this approach and investigates oribatid passive dispersal using rodents in restored heathlands of different age. We measured the number of oribatid mites in the pelt of rodents and movements of the latter, thus assessing the transport capacity of small mammals for oribatid mite dispersal. We also identified oribatid species and measured their abundance in rodents' nests to study whether the oribatids can survive there and move from one individual rodent to another, thus increasing their dispersal distance.

## 2 MATERIALS AND METHODS

### 2.1 Study area

The study was carried out on the edges of a sand pit in an extensive heathland area in the National Park Hoge Kempen, in the eastern part of Belgium (50°58′13.8″N, 5°38′11.1″E). From the 1960s onwards the pit was enlarged in a western direction leaving behind an artificial lake with edges of different ages since excavation. Heathlands were restored along these edges, creating a set of sites of known age. We selected two sites largely dominated by Common heather (*Calluna vulgaris* (L.) Hull). One site (50°58′23.48″N, 5°38′22.55″E) was restored into heathland in the 1980s and referred to as the restored site. This site of over 30 years consisted of a parcel of approximately 1 ha dominated by *C. vulgaris*, borders a pine (*Pinus* spp.) forest in the north, and contains some patches of the shrub Common broom (*Cytisus scoparius var. scoparius* (L.) Link) and a few young pine trees. The second is a heathland site that is at least 100 years old (50°58′38.8″N 5°38′22.1″E) which lies approximately 600 m north of the restored site and also consists of an approximately 1 ha parcel. This site was selected as a reference and is also dominated by *C. vulgaris*. At a larger scale (1–3 km), the sites are surrounded by a mix of deciduous forest and pine plantations that are in the process of being converted into heathland.

### 2.2 Travel distance

The movement of small rodents was estimated using two non‐exclusive and complementary methods: telemetric tracking and capture–mark–recapture (CMR). These methods were applied to estimate straight distances traveled per hour and to validate well‐documented patterns in both wood mice (*Apodemus sylvaticus* (Linnaeus, 1758)) and bank voles (*Myodes glareolus* (Schreber, 1780)) locally (Bergstedt, 1966; Geuse et al., 1985; Tolkachev, 2016). For the CMR campaign, 120 non‐lethal Sherman traps (Sherman Live Trap Co.) were checked recurrently during daylight hours for 3 consecutive days. This led to a total of captured 189 individuals, and from these 38 were captured more than once. Their range of movement was estimated by measuring the distance between the most widely separated capture points (Gurnell & Flowerdew, 2006). Using this data, the size of their populations was estimated using the Schnabel method (mean Petersen estimate; Schnabel, 1938). For telemetric tracking, we used 40 traps per site in two different sites (i.e., 80 in total). Once captured, 14 individuals were tagged with collar transmitters (BD‐2C transmitters for mouse/vole – HSL). These were tracked using a receiver (Model R410‐ATS) with a three‐element folding Yagi antenna (151Mhz‐ATS) for medium range (5–80 m). Rodents were tracked during active hours (i.e., from sunset to sunrise) using triangulation methods for 15 days and accounted for a total of 80 points in time (White & Garrott, 1990), as movement tracking was frequently interrupted by labor‐intensive tracking and digging of nests. Using the coordinates recorded, we estimated movement events as straight‐line segments (or steps; Bovet & Benhamou, 1988). Distances traveled by rodents recorded using both tracking methods (i.e., telemetry and capture–mark–recapture) were, respectively, plotted using a diagram of relative movements paths standardized to the (0,0) origin (QGIS 3.14.15), and a histogram plotting the number of individuals traveling different distances.

### 2.3 Sample collection

**FIGURE 1:**
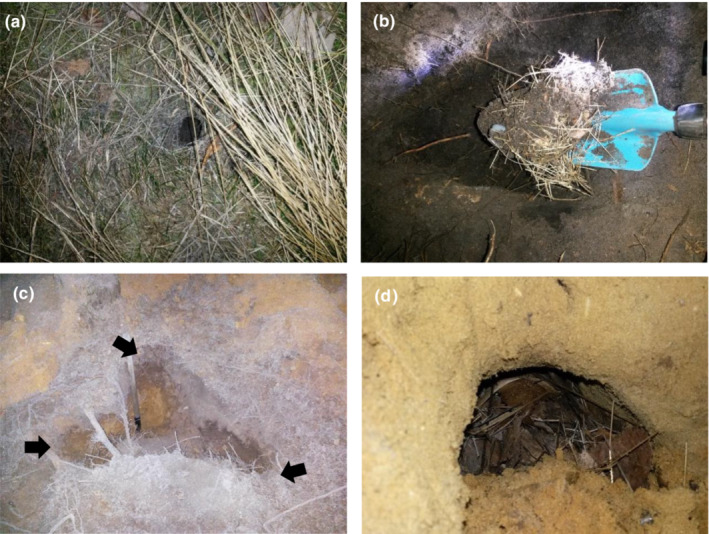
Nests of wood mice varying in depth. Restored site: Single entrance—30 cm depth—(a) and its content (b), and reference site: Excavated burrow with three entrances indicated with arrows—90 cm depth—(c) and its content (d).

Mite samples were collected from three different sources: pelt of rodents, nests of rodents, and soil. The sampling of pelts took place at the end of each month in 2015 between February and October. During each sampling period, 120 non‐lethal Sherman traps (Sherman Live Trap Co.) were checked recurrently during daylight hours for 3 consecutive days. A total of 189 rodents were caught in the traps and checked for the presence of arthropods using a small brush (details in the following section). On this campaign samples were collected in restored heahtlands to assess whether oribatid mites were mounting rodents. Based on the positive results of the latter, nests were collected in a second campaign during winter (December 2016 and February 2017) by tracking small rodents tagged using collar transmitters, and a reference site was included to compare the effects of succession on the probabilities of phoretic inteactions between rodents and oribatid mites. We used a receiver (Model R410‐ATS) with a modified short‐range antenna (0–5 m) to detect the underground signals. The extraction of the nests was carried out in burrows with a static signal during inactive hours (broad daylight). Only nests on which the transmitters were found or from which the rodents ran out while digging were extracted in order to be certain that these belonged to the tagged specimens. A total of 12 nests inside burrows varying in depth (i.e., 15–90 cm) and consisting of leaves, litter, and other nesting material were collected (Figure 1): six in the restored site and six in the reference: each nest was considered a whole sampling unit independently from its size. Soil samples from the top layer of the soil (0–15 cm) were collected from the proximity of each nest (i.e., 12 soil samples in total), at approximately 50 cm from the nest entrance; soil samples were approximately 100 cm^3^. This was done to compare oribatid community composition in rodent nests and the surrounding soil.

### 2.4 Mite extraction and identification

We extracted the arthropods from the soil and nest samples with a modified Tullgren apparatus lit with 60 W incandescent bulbs to collect them in 70% ethanol. The temperature of the apparatus was gradually increased over a period of 7 days to decelerate the desiccation of soil and allow species with different humidity preferences to move downward, i.e., starting at 20°C, 30°C on day 3, and 56°C on day 5 (Crossley & Blair, 1991; Edwards, 1991). We then separated the oribatid mites and cleansed each sample in a small tube with 4 ml 70% lactic acid solution for 2 days.

For pelt samples, every captured rodent was identified to species level and brushed over a white bowl using a small brush to check for the presence of oribatids. The brush consisted of three rows of densely distributed bristles (approximately 1 mm of separation) with 1.5 cm of length and covering a surface of 4 cm x 2 cm. To maximize this extraction, rodents were thoroughly and repeatedly combed in the direction of the hair growth for approximately 60 s. All material originating from the pelt of these mice was then transferred into Eppendorf tubes with 70% ethanol. Except for an individual (*Dissorhina ornata* (Oudemans, 1900)) that appeared to be partially eaten by a predatory mite, all specimens were in good condition and thus seemingly alive during their extraction. Only adult mites were identified to the species level following Weigmann (2006) and then stored in a glycerol medium. Habitat preferences, i.e., soil dwelling, arboreal, wandering (i.e., between different habitats), and nidicolous, and reproduction mode, i.e., sexual and parthenogenetic, were assigned following Bukva et al. (1976), Weigmann (2006), Subías (1977), Travé (1963), Behan‐Pelletier and Winchester (1998), Krivolutsky (2004), Winchester et al. (1999), and Maraun et al. (2009).

### 2.5 Statistical analysis

Differences in the distances traveled by rodents were tested using type II sum of squares on a general linear model (GLM) after dropping interactions (non‐significant; Langsrud, 2003; R Core Team v.3.4.1, 2017). Three independent variables were treated as categorical factors, i.e., site (two levels, restored and reference), rodent species (two levels, wood mice and bank vole), and tracking method (two levels, telemetry and CMR). The annual mite dispersal (AMD) was estimated by multiplying the potential mites carried (PMC) and the daily nest visits (DNV). The latter was estimated by adapting models used on pollen and fungi dispersal (Muñoz‐Adalia et al., 2017; Ne'eman et al., 1999). Both parameters Ah (time active per day) and V (time spent out of nests) used to estimate DNV were based on literature (Corp et al., 1997; Wolton, 1983, 1985). The equations for these estimations are presented below (Equations (1), (2), (3)).

Equation 1. Potential mites carried. Variables: *R* (range of rodents in population), *M* (range of mites dispersed per rodent), and Fr (frequency of mites found on rodents).

(1) $$\mathrm{PMC}=\left(\sum_{i}^{n}R\right)\left(\sum_{i}^{n}M\right)\left(\mathrm{Fr}\right)$$

Equation 2. Daily nest visits. Variables: *Ah* (range of time active of rodents per day ‐hours‐) and *V* (range of time spent out of nests per day ‐hours‐).

(2) $$\mathrm{DNV}=\frac{\sum_{i}^{n}\mathrm{Ah}}{\sum_{i}^{n}V}$$

Equation 3. Annual mite dispersal. Variables: PMC (potential mites carried) and DNV (daily nest visitations).

(3) $$\mathrm{AMD}=\left(\mathrm{PMC}\right)\left(\mathrm{DNV}\right)\left(365\right)$$

To explore if the oribatid mite community transported by small mammals was different in sites of different successional ages and sources, we analyzed abundance and species richness in pelts, nests, and soil; species richness, i.e., mean and SD, was estimated on the presence/absence transformed data. We analyzed the effects of succession on the abundance and species richness using two separate negative binomial generalized linear models with a single independent variable (i.e., abundance or species richness), and treating site type (two levels, restored and reference) and source (two levels, nest and soil) as categorical factors in both models. This type of model was selected to address the excess of zeros leading to highly overdispersed data, and after failing fit tests using Poisson and quasi‐Poisson distribution (Obiegala et al., 2021). Back‐transformed rate ratios (*β*), standard error (SE), and *p*‐value (*p*) were reported for significant differences. Interactions between main effects were evaluated for all tests, but main effects are only reported when the interactions were not significant; in the latter case, the interactions are dropped from the models. For pelt samples (*N* = 189), only abundance and occurrence probabilities were estimated, and separately from nest (*N* = 12) and soil (*N* = 12) samples due to the differences in sampling size and collection methods. Patterns in the community composition were also analyzed with a NMDS ordination using log‐transformed relative abundances.

## 3 RESULTS

### 3.1 Phoresy probabilities

Oribatids were present in over 6% of the pelt samples (*N* = 189), 92% of the rodent nests (*N* = 12), and 58% of the soil samples (*N* = 12). Individuals in pelts, nests, and soil in our study, respectively, accounted for a total of 14, 613, and 203. In terms of abundances, 1–2 oribatid mites were often found per host, and the population size of rodents in 1 ha sites ranged between 13 and 75 individuals throughout the year. This results in 2–10 oribatid mites being carried (PMC) by the local rodent population at any given time. Considering that behavioral studies have shown that small rodents may visit their nest every 50 min but may take up to 8 h per outing, and that they may remain active between 1 and 8.5 h depending on the season, we estimated nest visitations per day (DNV) to range between 2 and 10. As a result, a minimum of 2 and a maximum 112 oribatid mites may be transported phoretically via rodents per day, thus 730–40,880 per year (AMD).

### 3.2 Oribatid communities

A total of 830 oribatid mites from 47 species and 21 families were identified (Appendix 1: Table A1). From these, nearly 70% of the specimens were collected from nests in the reference site. The reference site was characterized by higher oribatid abundances (*β* = 13.6, SE = 0.6, *p* < .001) and species richness (*β* = 3.7, SE = 0.4, *p* < .001; Table 1). None of the metrics showed significant differences between nest and soil samples (*β* = 0.3, SE = 0.6, *p* = −1.9 and *β* = 0.8, SE = 0.4, *p* = −.4, respectively, for abundance and species richness); pelt samples were analyzed separately due to the differences in sampling size and collection methods.

**TABLE 1:** Mean, standard deviation (SD), standard error (SE), and total numbers for abundance and species richness of different sources and sites.

Site	Reference		Restored		
Source	Nest	Soil	Nest	Soil	Pelt
*n*	12	12	12	12	189
Abundance					
Total	571	189	42	14	14
Mean (SD)/100 cm^3^	95.2 (140.9)	31.5 (47.1)	7 (4.8)	2.3 (3.8)	0.1 (0.3)
SE	57.5	19.2	1.9	1.6	0.02
Sp. Richness					
Total	30	30	8	7	10
Mean (SD)	7 (5.1)	7.5 (7.2)	2.5 (1.2)	1.5 (2.3)	0.05 (0.1)
SE	2.1	3	0.5	1	0.01

*Note*: Pelt samples were only collected in the restored site and at a different sampling campaign.

**FIGURE 2:**
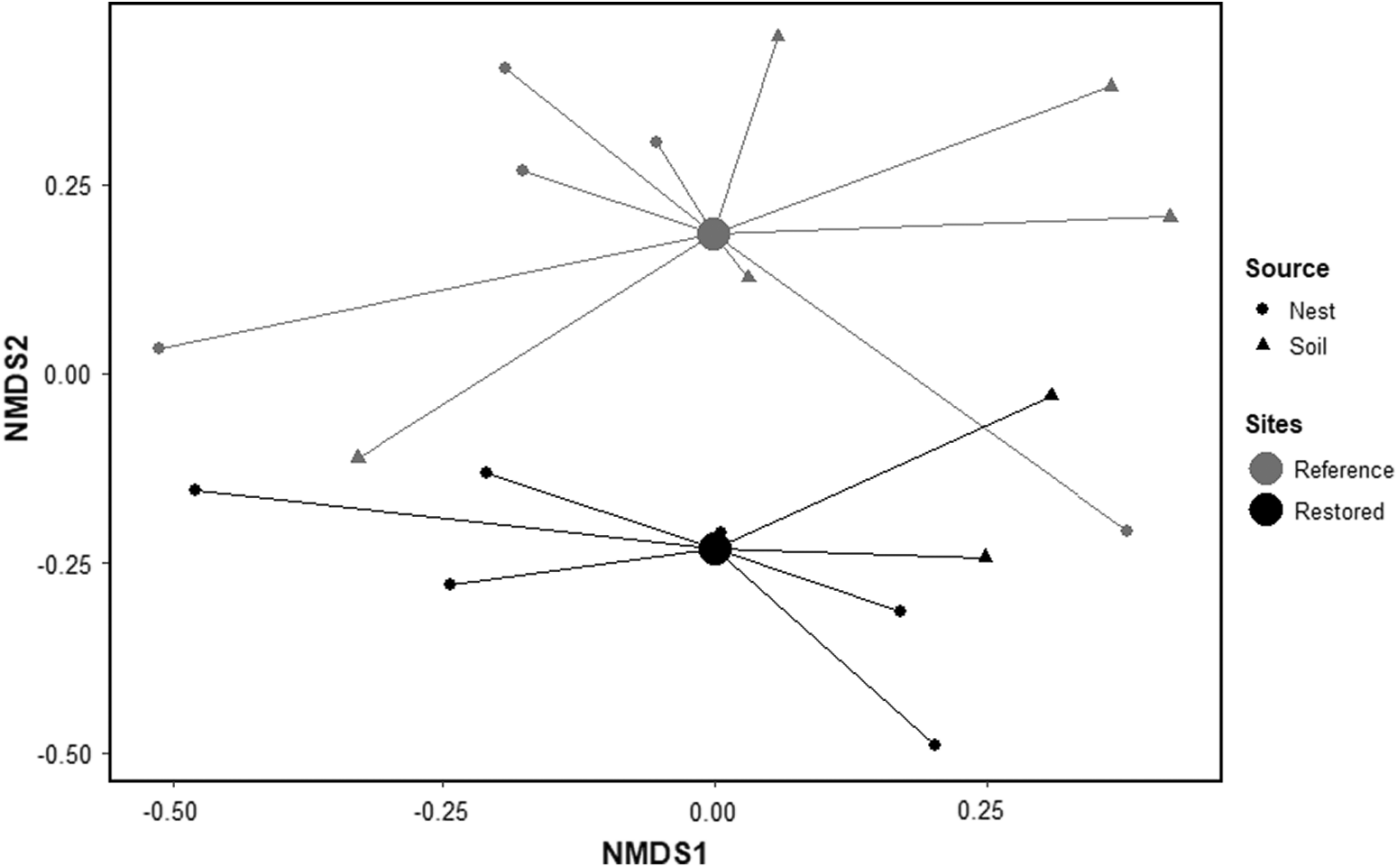
NMDS ordination on log‐transformed relative abundances of oribatid mites. Nest (*n* = 11) and soil samples (*n* = 7) are, respectively, plotted using circles and triangles, and sites are indicated using both spider diagrams connecting each point to the centroid of its respective site and color differences: Reference (gray) and restored (black). A total of six samples with zero individuals were excluded from the NMDS analysis.

The habitat preference of the oribatid species in our study was primarily wandering (51%), followed by soil dwelling (32%), arboreal (15%), and nidicolous (2%), and over half of the species reproduced asexually (58%). Aside from an extreme value of *Oribella pectinata* (Michael, 1885) accounting for more than half (56%) of the specimens, *Oppiella nova* (Oudemans, 1902) (20%) and *Microtritia minima* (Berlese, 1904) (5%) were the most abundant species. *Microtritia minima*, *O. nova*, and *M. minus* were particularly prevalent in the restored site. In pelt, specimens from the genus *Chamobates* (Hull, 1916) were most abundant (31%), followed by *Diapterobates humeralis* (Hermann, 1804; 13%) and nine other species accounting for 6% each. In soil samples, the species were more evenly distributed, with the highest abundance of 11% for both *M. minus* and *Trichoribates trimaculatus* (Koch, 1835), and 9% for *Acrotritia duplicata* (Grandjean, 1953), *Nothrus silvestris* (Nicolet, 1855), and *Dissorhina ornata* (Oudemans, 1900) each. These differences led to a clear discrimination between sites, i.e., reference and restored in the NMDS ordination, but clusters of nests and soil samples were less pronounced (Figure 2). Compared to sites, the distribution of nests was more scattered in space showing that the species pool in these microsites is broad and independent from successional age.

### 3.3 Dispersal range

**FIGURE 3:**
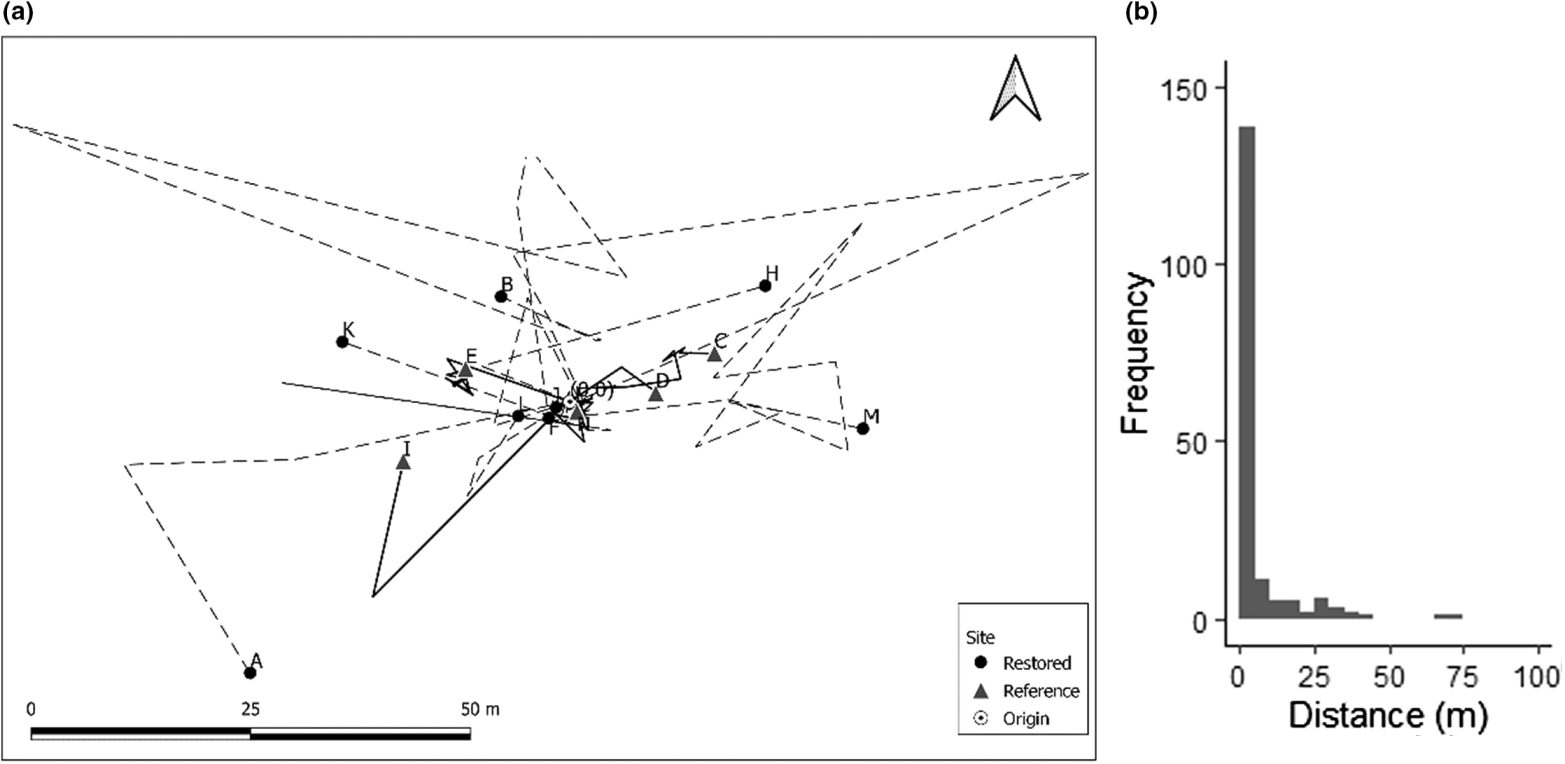
(a) Movement trajectories from telemetric tracking standardized to the (0,0) origin for a total of 80 fixes recorded from 8 rodents in the restored (black dot and dotted line) and 5 in the reference sites (gray triangles and solid line), respectively, accounting for 46 and 34 fixes. (b) Histogram of movement using capture–mark–recapture method. From a total of 189 individuals, 151 were captured only once leading to zero in the distance traveled, and the distances for the remaining 38 individuals captured more than once were estimated as the distances between the most widely separated capture points.

From the 14 rodents tracked using the telemetric approach, 13 were recorded in at least two points in space and accounted for a total of 80 fixes (steps; Figure 3a), and 38 of 189 individuals of the capture–mark–recapture campaigns were captured more than once (Figure 3b). Nearly 90% of the movement events were below 40 m and only a few individuals almost reached a 100 m between the farthest points recorded. Rodents from the restored site clearly moved larger distances than those from the reference site (AOV: *F*_1,114_ = 18.1, *p* < .001). Wood mice were the dominant species (65%) caught in the study and traveled over larger distances than bank voles (AOV: *F*_1,114_ = 10, *p* < .01). The distances estimated from the capture–mark–recapture method and the telemetric data showed no significant differences (AOV: *F*_1,114_ = 0.6, *p* = .45).

## 4 DISCUSSION

Compared to other reported hosts such as birds and larger arthropods (Appendix 1: Table A2), small rodents and their nests contained similar amounts of oribatid mites but the frequencies of hosts carrying mites were lower. For instance, the numbers of birds carrying mites were much higher (>60%), and moderately higher in arthropods (7%–16%; Knee et al., 2013; Norton, 1973); only single events reported for herpetofauna prevented from retrieving group frequencies (Beaty et al., 2013; Mendoza‐Roldan et al., 2020). However, our results indicate that rodents may aid the dispersal of over 40,000 specimens per year per ha. Whereas this number may seem low for a group with densities typically ranging between 20,000 and 50,000 per square meter in heathlands (Frouz et al., 2009; Webb, 1994), the slow turnover rate of oribatid communities in such nutrient‐poor ecosystems makes these modest values relevant in the long term (Kardol et al., 2009; Salazar‐Fillippo et al., unpublished data; Zaitsev et al., 2006). In the three to five decades required to reach approximately stable oribatid communities (Ryabinin & Pan'kov, 2009; Scheu & Schulz, 1996), annual values of 40,000 would imply an influx of 1–2 million individuals into the area.

Interestingly, both bank voles and wood mice traveled longer distances in the restored site. This contradicts our expectation that higher vegetation cover in developed heathlands i.e., reference site would favor movement (Spirito et al., 2020). In a more extreme comparison, however, Corp et al. (1997) reported that the lower food availability in sand dunes led to higher activity, movement speeds, and distances traveled as compared to more productive woodlands. Similar behavioral differences might also occur in our heathland sites where food resources are most likely more scarce in sites of earlier successional stages. The young ‐restored‐ site also had a higher presence of bushes that may provide additional protection against predators. This may lead to more distant‐ranging events (Fox et al., 2003) and is particularly important because rodents prefer such dense bushes to establish their nests (Rosalino et al., 2011). Moreover, both methods used to track the movement of rodents (i.e., telemetric triangulation and capture–recapture) showed mean distances above 20 m and maxima of nearly 100 m. Other reports on the movement range of small rodents come to similar values (Bergstedt, 1966; Geuse et al., 1985; Tolkachev, 2016), and we observed within these ranges that small rodents could nest in groups (i.e., up to three rodents in the same burrow) and several individuals had more than one nest. Additionally, small rodents in heathlands may change nesting locations frequently and use burrows of other species (Benhamou, 1990; Wolton, 1983, 1985), all of which may enhance oribatid dispersal. Moreover, there is also an inverse relationship between similarity in community composition after restoration and distance to source populations (van der Bij et al., 2018), most likely because dispersal from neighboring sites is more frequent than from sites further away (van Diggelen et al., 2021).

Our results on species composition both in pelts and nests of rodents support the relevance of this group for the assembly of oribatid communities. While 10 of 14 oribatid mites in the pelts belonged to different species, and 70% of these were also present in nests, rodents appear to host a broad species pool of oribatid mites—unlike dispersal vectors like wind or water which are highly filtering specific species (Lehmitz et al., 2011; Schuppenhauer et al., 2019). Albeit diversity metrics showed no significant differences between sources, similarities in the community composition of oribatids in nests observed in the NMDS plot suggest that rodents transport oribatids from nest to nest regardless of site age. In 12 sampled nests—compared to 189 rodents—we found 31 species belonging to 15 families, making it highly likely that our sample size fails to represent the whole community composition here. The latter is highly likely as Bukva et al. (1976) reported over 70 species in a more extensive survey in nests of rodents. Nests may thus contribute to the local species pool by functioning as buffers assisting the colonization of the soils surrounding the nests by species capable of withstanding local environmental conditions.

The diversities of oribatid mites on small mammals and their nests are higher than in most other host groups, and only studies about birds and their nests have reported higher diversities (Ermilov et al., 2013; Krivolutsky & Lebedeva, 2004; Lebedeva, 2007; Pilskog et al., 2014). From these, species with a parthenogenetic reproductive mode were dominant (58%) and a third of the community composition presented a soil‐dwelling lifestyle. The latter is particularly relevant for succession in restored landscapes because of the important role oribatid mites play in the decomposition of organic matter (Norton, 1984; Siepel & Maaskamp, 1994). Despite its ecological importance (Karasawa & Hijii, 2008; Schneider et al., 2004), most authors do not mention habitat preferences, except for Krivolutsky and Lebedeva (2004) and Lehmitz et al. (2011) who report the dominance of arboreal species, respectively, in bird nests and wind as the main dispersal vector. Most studies on phoresy of oribatid mites emphasize the impact of asexual reproduction on the assembly of oribatid communities (Coulson, 2009; Lebedeva, 2007), as it ensures that rare phoretic events may lead to a successful colonization (Norton & Palmer, 1991). The most abundant species in nests had a relatively small size (<450 μm), presumably because small animals are easier to transport (Lehmitz et al., 2011; Schuppenhauer et al., 2019). Unfortunately, the small sample size prohibited a statistical analysis of trait patterns to test such hypotheses as in Salazar‐Fillippo et al., unpublished data. Further trait‐based research may provide critical input regarding the role of guilds (e.g., follower or facilitator) during succession (Hobbs et al., 2009).

Although our literature review on oribatid transport via different types of hosts is not complete, only nine of 35 studies provided numerical assessments of phoretic transport or enough data to estimate such values. This shows important research gaps on the role of phoresy in the assembly of oribatid communities and, more importantly, its potential application. Restoration ecology, for instance, has gradually become more focused on the role of belowground communities as essential components of the ecosystem. Phoresy represents an understudied subject that, given the long‐term timeframe of restoration, could provide essential insight into the structuring and functioning of belowground communities and could eventually be translated into practical management approaches.

## AUTHOR CONTRIBUTIONS

**Andrés A. Salazar‐Fillippo:** Conceptualization (equal); data curation (lead); formal analysis (lead); investigation (equal); methodology (equal); software (lead); writing – original draft (lead). **Bert Teunkens:** Data curation (equal); formal analysis (supporting); investigation (equal); methodology (equal); software (supporting). **Herwig Leirs:** Conceptualization (equal); funding acquisition (equal); investigation (equal); methodology (equal); project administration (equal); resources (equal); supervision (equal); validation (equal). **Jan Frouz:** Conceptualization (equal); funding acquisition (equal); investigation (equal); methodology (equal); supervision (equal); validation (equal); visualization (equal); writing – review and editing (equal). **Rudy van Diggelen:** Conceptualization (equal); funding acquisition (equal); investigation (equal); project administration (lead); supervision (lead); validation (equal); visualization (equal); writing – original draft (supporting); writing – review and editing (lead). **Ladislav Miko:** Conceptualization (equal); data curation (equal); investigation (equal); methodology (equal); supervision (lead); validation (equal); writing – review and editing (lead).

## CONFLICT OF INTEREST

The authors declare that they have no known competing financial interests or personal relationships that could have appeared to influence the work reported in this study.

## Data Availability

Data have been archived in the Dryad Digital Repository doi: 10.5061/dryad.mgqnk9935 (Salazar‐Fillippo et al., unpublished data).

## APPENDIX 1

**TABLE A1:** The abundance of oribatid mites according to site and substrate

No.	Species	Habitat Pref.	Rep. Mode	Reference		Restored		Soil	Total
				Nest	Soil	Nest	Pelt		
1	*Oribella pectinata* (Michael, 1885)	Nest	S	55.5 (124.4)	0.7 (1.2)	1.5 (1.4)	0.1 (0.3)	0 (0)	347
2	*Oppiella nova* (Oudemans, 1902)	Wandering	P	20.5 (22.5)	2.5 (5.6)	0.3 (0.8)	0.1 (0.3)	0 (0)	139
3	*Microtritia minima* (Berlese, 1904)	Soil	P	1.7 (4.1)	0 (0)	3.8 (4.8)	0 (0)	0.8 (2)	38
4	*Microppia minus* (Paoli, 1908)	Wandering	P	0.3 (0.8)	3.3 (6.7)	0.7 (1.2)	0 (0)	0.5 (0.8)	29
5	*Trichoribates trimaculatus* (Koch, 1835)	Arboreal	S	0.2 (0.4)	3.7 (9)	0 (0)	0.1 (0.3)	0 (0)	24
6	*Rhysotritia duplicata* (Grandjean, 1953)	Soil	P	0.8 (1.6)	3 (5.3)	0 (0)	0 (0)	0 (0)	23
7	*Moritzoppia unicarinata* (Paoli, 1908)	Soil	S	3.5 (8.6)	0 (0)	0 (0)	0 (0)	0 (0)	21
8	*Nothrus silvestris* (Nicolet, 1855)	Wandering	P	0.3 (0.5)	3 (6.9)	0 (0)	0 (0)	0 (0)	20
9	*Tectocepheus v. velatus* (Michael, 1880)	Wandering	P	0.5 (1.2)	2.7 (2.3)	0.2 (0.4)	0 (0)	0 (0)	20
10	*Dissorhina ornata* (Oudemans, 1900)	Arboreal	S	0 (0)	3 (7.3)	0 (0)	0.1 (0.3)	0 (0)	19
11	*Suctobelbella arcana* (Moritz, 1970)	Wandering	P	2.2 (3.4)	0.5 (0.8)	0 (0)	0 (0)	0.3 (0.5)	18
12	*Suctobelbella subtrigona* (Oudemans, 1900)	Wandering	P	0.7 (1.6)	1.7 (4.1)	0 (0)	0 (0)	0 (0)	14
13	*Chamobates pusillus* (Berlese, 1895)	Wandering	S	0.8 (1.3)	0.7 (1.6)	0 (0)	0.2 (0.4)	0 (0)	12
14	*Scheloribates latipes* (Koch, 1844)	Wandering	S	0.8 (1.6)	1.2 (1.3)	0 (0)	0 (0)	0 (0)	12
15	*Multioppia laniseta* (Moritz, 1966)	Soil	S	1.7 (4.1)	0 (0)	0 (0)	0 (0)	0 (0)	10
16	*Cultroribula bicultrata* (Berlese, 1905)	Wandering	P	1.2 (2.9)	0.2 (0.4)	0 (0)	0 (0)	0 (0)	8
17	*Odontocepheus elongatus* (Michael, 1879)	Arboreal	S	0.5 (0.8)	0.2 (0.4)	0.2 (0.4)	0.1 (0.3)	0.2 (0.4)	7
18	*Moritzoppia translamellata* (Willmann, 1923)	Soil	S	1 (2.4)	0 (0)	0 (0)	0 (0)	0 (0)	6
19	*Eniochthonius minutissimus* (Berlese, 1904)	Soil	P	0.5 (1.2)	0.3 (0.8)	0 (0)	0 (0)	0 (0)	5
20	*Oppiella splendens* (C. L. Koch, 1841)	Soil	P	0 (0)	0.8 (2)	0 (0)	0 (0)	0 (0)	5
21	*Suctobelbella acutidens* (Forsslund, 1941)	Wandering	P	0.3 (0.8)	0.3 (0.8)	0 (0)	0 (0)	0.2 (0.4)	5
22	*Damaeus clavipes* (Hermann, 1804)	Soil	S	0 (0)	0.7 (1.6)	0 (0)	0 (0)	0 (0)	4
23	*Diapterobates humeralis* (Hermann, 1804)	Arboreal	S	0.3 (0.8)	0 (0)	0 (0)	0.1 (0.4)	0 (0)	4
24	*Suctobelbella falcata* (Forsslund, 1941)	Wandering	P	0.3 (0.8)	0.3 (0.5)	0 (0)	0 (0)	0 (0)	4
25	*Oribatula tibialis* (Nicolet, 1855)	Wandering	S	0.2 (0.4)	0.3 (0.8)	0 (0)	0 (0)	0 (0)	3
26	*Pergalumna nervosa* (Berlese, 1914)	Soil	P	0.2 (0.4)	0.2 (0.4)	0.2 (0.4)	0 (0)	0 (0)	3
27	*Suctobelbella similis* (Forsslund, 1941)	Wandering	P	0 (0)	0.5 (1.2)	0 (0)	0 (0)	0 (0)	3
28	*Suctobelbella subcornigera* (Forsslund, 1941)	Wandering	P	0 (0)	0.5 (0.5)	0 (0)	0 (0)	0 (0)	3
29	*Berniniella sigma* (Strenzke, 1951)	Wandering	S	0.3 (0.8)	0 (0)	0 (0)	0 (0)	0 (0)	2
30	*Camisia spinifer* (Koch, 1835)	Arboreal	P	0 (0)	0 (0)	0 (0)	0.1 (0.3)	0.2 (0.4)	2
31	*Ceratoppia quadridentata* (Haller, 1882)	Wandering	S	0 (0)	0.3 (0.8)	0 (0)	0 (0)	0 (0)	2
32	*Chamobates* sp. (Hull, 1916)	Wandering	S	0 (0)	0 (0)	0 (0)	0.1 (0.4)	0 (0)	2
33	*Suctobelbella palustris* (Forsslund, 1953)	Wandering	P	0.3 (0.5)	0 (0)	0 (0)	0 (0)	0 (0)	2
34	*Adoristes ovatus* (Koch, 1839)	Wandering	S	0 (0)	0 (0)	0.2 (0.4)	0 (0)	0 (0)	1
35	*Banksinoma lanceolata* (Michael, 1885)	Arboreal	S	0.2 (0.4)	0 (0)	0 (0)	0 (0)	0 (0)	1
36	*Chamobates borealis* (Trägårdh, 1902)	Wandering	S	0 (0)	0.2 (0.4)	0 (0)	0 (0)	0 (0)	1
37	*Dometorina plantivaga* (Berlese, 1895)	Arboreal	S	0 (0)	0 (0)	0 (0)	0.1 (0.3)	0 (0)	1
38	*Euphthiracarus cribarius* (Berlese, 1904)	Soil	P	0 (0)	0 (0)	0 (0)	0 (0)	0.2 (0.4)	1
39	*Hypochthonius luteus* (Oudemans, 1917)	Wandering	P	0 (0)	0.2 (0.4)	0 (0)	0 (0)	0 (0)	1
40	*Hypochthonius rufulus* (Koch, 1835)	Soil	P	0 (0)	0.2 (0.4)	0 (0)	0 (0)	0 (0)	1
41	*Nothrus borussicus* (Sellnick, 1928)	Soil	P	0.2 (0.4)	0 (0)	0 (0)	0 (0)	0 (0)	1
42	*Ophidiotrichus tectus* (Michael, 1884)	Soil	S	0 (0)	0.2 (0.4)	0 (0)	0 (0)	0 (0)	1
43	*Phthiracarus longulus* (Koch, 1841)	Soil	P	0.2 (0.4)	0 (0)	0 (0)	0 (0)	0 (0)	1
44	*Steganacarus spinosus* (Sellnick, 1920)	Soil	P	0 (0)	0.2 (0.4)	0 (0)	0 (0)	0 (0)	1
45	*Tectocepheus alatus* (Berlese, 1913)	Wandering	P	0.2 (0.4)	0 (0)	0 (0)	0 (0)	0 (0)	1
46	*Tectocepheus v. sarekensis* (Trägårdh, 1910)	Wandering	P	0.2 (0.4)	0 (0)	0 (0)	0 (0)	0 (0)	1
47	*Trichoribates novus* (Sellnick, 1928)	Wandering	S	0 (0)	0.2 (0.4)	0 (0)	0 (0)	0 (0)	1

*Note*: Mean values and standard deviation are given. Habitat preference (levels: Nest, Soil, Wandering, Arboreal) and reproduction mode (levels: Sexual, Parthenogenetic) follow Bukva et al. (1976), Weigmann (2006), Subías (1977), Travé (1963), Behan‐Pelletier and Winchester (1998), Krivolutsky (2004), Winchester et al. (1999), and Maraun et al. (2009). Taxonomy follows Weigmann (2006) and species are ordered according to their total abundance.

**TABLE A2:** Phoretic interactions between oribatid mites and reported host groups

Group	Host		Oribatid mites		Interaction (mites/host)			
	∑	Freq.	Species	∑	Average^a^	Max.	Species	Reference
Current study								
Soil	12	58.3%	41	203	29 ± 43.4	123	3–21	
Rodents	189	6.3%	10	14	1.1 ± 0.4	2	1–2	
Rodent nests	12	92%	31	613	55.7 ± 109.4	374	1–14	
Population sample								
Birds								
Passerine	241	≅66%	76		1–2	21	1–26	Lebedeva and Lebedev (2008)
Passerine/Corvidae	1222		61		1–17		1–61	Krivolutsky and Lebedeva (2004)
Raptors		≅66%	≅60		2.2 ± 3	13	1–14	Lebedeva (2007)
Not specified			78					Lebedeva and Poltavskaya (2013)
Nests			64					Lebedeva and Poltavskaya (2013)
	21		53					Lebedeva and Lebedev (2008)
	63		79		1–275		1–41	Krivolutsky and Lebedeva (2004)
	17	100%	39	1180	3–216	216	2–21	Ermilov et al. (2013)
			17					Pilskog et al. (2014)
Review			≅60		1–2	21	1–26	Lebedeva (2010)
Arthropods								
Beetles	5635	16.4%^b^	2	194	2.5 ± 1.7^b^			Knee et al. (2013)
			1					Ermilov and O'connor (2020)
			6					Ahadiyat and Akrami (2015)
	5		5	149			1	Ermilov and Frolov (2019b)
	60		2	3				Pernek et al. (2012)
	6		1	6	1	1	1	Pernek et al. (2008)
			2	414				Penttinen et al. (2013)
	28	7,1%			4.5 ± 3.5			Norton (1973)
Bees			4		1–2	14		Sumangala and Haq (2001)
Review			22	2014	1–504	504	1–3	Norton (1980)
Mammals								
Rodents		6.1%	42					Miko and Stanko (1991)
	1863	2.5%		85				Vesotskaya and Bulanova‐Zaxvatkina (1957)
Rodent nests	16		6	87				Dubinina et al. (1966)
	303			13,364				Kramárová and Mrciak (1973)
	13		7	159				Costa (1961)
	298	70,5%		6596				Vesotskaya and Bulanova‐Zaxvatkina (1957)
	278	44.6%	72	979	1–5	72	1–5	Bukva et al. (1976)
	44		11	326				Krawczyk et al. (2015)
Single host report								
Amphibian	1		1	85		85	1	Mendoza‐Roldan et al. (2020)
	1		1	≅100		≅100	1	Beaty et al. (2013)
Dipteran	1		1	5		5	1	Coulson (2009)
Beetles	1		1	13		13	1	Ermilov (2019)
	1		1	≅200		≅200	1	Ermilov and Frolov (2019a) and Ermilov and Frolov (2019b)
Hemiptera	1		1	12		12	1	Waleckx et al. (2018)
Harvestman	1		1	≅30		≅30	1	Townsend et al. (2008)

*Note*: ≅ is used whenever the authors provide rough estimates or the total numbers are unclear.

^a^ Average: Mean ± SD presented when available, otherwise typical ranges provided in the articles were used.

^b^ Authors estimated these metrics including both oribatida and mesostigmata.
